# Attaining sinus rhythm mediates improved outcome with early rhythm control therapy of atrial fibrillation: the EAST-AFNET 4 trial^[Author-notes ehac471-FM2]^

**DOI:** 10.1093/eurheartj/ehac471

**Published:** 2022-08-29

**Authors:** Lars Eckardt, Susanne Sehner, Anna Suling, Katrin Borof, Guenter Breithardt, Harry Crijns, Andreas Goette, Karl Wegscheider, Antonia Zapf, John Camm, Andreas Metzner, Paulus Kirchhof

**Affiliations:** Department of Cardiology II (Electrophysiology), University Hospital Münster, Münster, Germany; Atrial Fibrillation Network (AFNET), Münster, Germany; Institute of Medical Biometry and Epidemiology, University Medical Center Hamburg–Eppendorf, Germany; Institute of Medical Biometry and Epidemiology, University Medical Center Hamburg–Eppendorf, Germany; Department of Cardiology, University Heart and Vascular Center, University Medical Center Hamburg–Eppendorf, Martinistraße 52, 20246 Hamburg, Germany; Department of Cardiology II (Electrophysiology), University Hospital Münster, Münster, Germany; Atrial Fibrillation Network (AFNET), Münster, Germany; Department of Cardiology, Maastricht University Medical Center and Cardiovascular Research Institute Maastricht, Maastricht, The Netherlands; Atrial Fibrillation Network (AFNET), Münster, Germany; Department of Cardiology, Vincenz-Krankenhaus Paderborn, Paderborn, Germany; Atrial Fibrillation Network (AFNET), Münster, Germany; Institute of Medical Biometry and Epidemiology, University Medical Center Hamburg–Eppendorf, Germany; DZHK (German Center for Cardiovascular Research), partner site Hamburg/Kiel/Luebeck, Berlin, Germany; Institute of Medical Biometry and Epidemiology, University Medical Center Hamburg–Eppendorf, Germany; DZHK (German Center for Cardiovascular Research), partner site Hamburg/Kiel/Luebeck, Berlin, Germany; Cardiology Clinical Academic Group, Molecular and Clinical Sciences Research Institute, St. George’s University of London, London, UK; Department of Cardiology, University Heart and Vascular Center, University Medical Center Hamburg–Eppendorf, Martinistraße 52, 20246 Hamburg, Germany; DZHK (German Center for Cardiovascular Research), partner site Hamburg/Kiel/Luebeck, Berlin, Germany; Atrial Fibrillation Network (AFNET), Münster, Germany; Department of Cardiology, University Heart and Vascular Center, University Medical Center Hamburg–Eppendorf, Martinistraße 52, 20246 Hamburg, Germany; DZHK (German Center for Cardiovascular Research), partner site Hamburg/Kiel/Luebeck, Berlin, Germany; Cardiovascular Sciences, University of Birmingham, Birmingham, UK

**Keywords:** Atrial fibrillation, Rhythm control, AF ablation, Antiarrhythmic drugs, Stroke, Randomized trials, Mediator analysis

## Abstract

**Aims:**

A strategy of systematic, early rhythm control (ERC) improves cardiovascular outcomes in patients with atrial fibrillation (AF). It is not known how this outcome-reducing effect is mediated.

**Methods and results:**

Using the Early treatment of Atrial Fibrillation for Stroke prevention Trial (EAST—AFNET 4) data set, potential mediators of the effect of ERC were identified in the total study population at 12-month follow up and further interrogated by use of a four-way decomposition of the treatment effect in an exponential model predicting future primary outcome events. Fourteen potential mediators of ERC were identified at the 12-month visit. Of these, sinus rhythm at 12 months explained 81% of the treatment effect of ERC compared with usual care during the remainder of follow up (4.1 years). In patients not in sinus rhythm at 12 months, ERC did not reduce future cardiovascular outcomes (hazard ratio 0.94, 95% confidence interval 0.65–1.67). Inclusion of AF recurrence in the model only explained 31% of the treatment effect, and inclusion of systolic blood pressure at 12 months only 10%. There was no difference in outcomes in patients who underwent AF ablation compared with those who did not undergo AF ablation.

**Conclusion:**

The effectiveness of early rhythm control is mediated by the presence of sinus rhythm at 12 months in the EAST-AFNET 4 trial. Clinicians implementing ERC should aim for rapid and sustained restoration of sinus rhythm in patients with recently diagnosed AF and cardiovascular comorbidities.

## Structured Graphical Abstract

**Structured graphical abstract:**
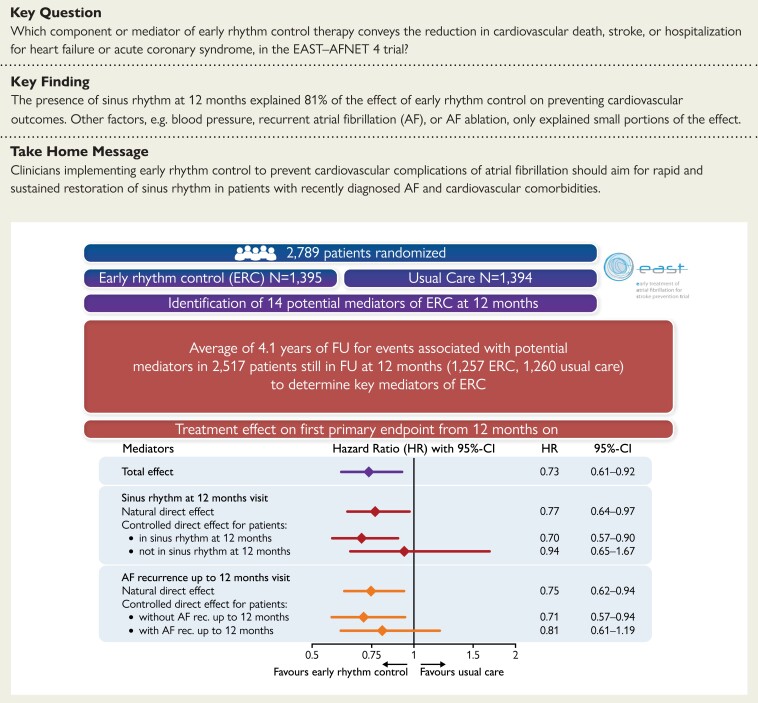
The EAST-AFNET 4 trial data set was analysed for potential mediators of the outcome-reducing effect of the early rhythm control strategy in the trial. All factors that were different between randomized groups at the 12-month visit were considered. Attaining sinus rhythm explained most of the treatment effect in a landmark analysis considering the remaining 4.1 follow-up years. In conclusion, clinicians aiming to implement early rhythm control should try to attain sinus rhythm.

**See the editorial comment for this article ‘Sinus rhythm: the** ***sine qua non*** **for rhythm control?’, by Dominik Linz and William F. McIntyre, https://doi.org/10.1093/eurheartj/ehac490.**

## Introduction

The Early treatment of Atrial Fibrillation for Stroke prevention Trial (EAST-AFNET 4) demonstrated that a strategy of systematic initiation of early rhythm control (ERC) reduced cardiovascular outcomes by 21% compared with usual care.^1^ Several prespecified subanalyses, including comparisons of patients with and without heart failure,^2^ with and without symptoms,^3^ and patients with different atrial fibrillation (AF) patterns,^4^ did not identify a differential effectiveness of ERC. The general safety of ERC has recently also been corroborated by analyses in large observational data sets.^5,6^ Based on the inclusion criteria of the EAST-AFNET 4 trial, the majority of patients with new-onset AF are eligible for ERC.^6^ The results have started to shift the use of rhythm control therapy from a symptom-driven therapy to a risk-reducing strategy aiming at restoring and maintaining sinus rhythm as the default therapy in patients with recently diagnosed AF and stroke risk factors.^7,8^

EAST-AFNET 4 tested ERC as a therapy strategy. There has been much speculation on the drivers of the reduction in outcomes within this treatment strategy. The availability of AF ablation, improvements in the safe use of antiarrhythmic drugs, including patients with heart failure, continuation of improved oral anticoagulation, and therapy of concomitant conditions irrespective of achieved rhythm,^9^ and the early initiation of rhythm control with the associated prevention of severe forms of atrial cardiomyopathy, have been discussed. To identify possible factors associated with prevention of cardiovascular outcomes on ERC therapy, we scrutinized the EAST-AFNET 4 trial data set for factors and mediators of ERC that are associated with reduced cardiovascular outcomes.

## Methods

Sample characteristics of the available patients are given as absolute and relative frequencies, mean ± standard deviation or as median with interquartile range. A two-tailed *P* < 0.05 was considered to be statistically significant. The reported *P*-values and confidence intervals (CIs) have not been adjusted for multiplicity. All analyses were conducted with Stata software (StataCorp 2021, Stata Statistical Software: Release 17; StataCorp LLC, College Station, TX, USA) and the package med4way.

For model building, missing values in medical relevant baseline variables as well as follow-up data of medication, recurrent AF, blood pressure, and secondary endpoints were multiply imputed with 65 repetitions following the recommendations of White *et al*.^10^ As in the primary analysis of the EAST trial, missing values were imputed in survivors only, except of the EQ-5D visual analogue scale where deceased patients received a score of 0.

All analyses were adjusted for medical relevant baseline variables to reduce potential confounding bias: age, gender, centre type (D or A site), European Heart Rhythm Association (EHRA) score, New York Heart Association (NYHA) class, prior stroke or transient ischaemic attack, AF pattern, left atrial diastolic diameter, MoCA (Montréal Cognitive Assessment) score, arterial hypertension, diabetes, peripheral artery disease, severe coronary artery disease (previous myocardial infarction, coronary artery bypass graft, or percutaneous coronary intervention), left ventricular hypertrophy on echocardiography (>15 mm wall thickness), left ventricular ejection fraction, AF duration at baseline (categorized <10, 10–100, and >100 days), chronic obstructive pulmonary disease, chronic kidney disease (Modification of Diet in Renal Disease Stage III or IV), physical and mental SF-12 component summary scores, and cardiac rhythm.

### Objective 1

A causal mediation analysis was conducted in the total study population to identify mediators of treatment success with respect to the primary outcome parameter and two of its key components, cardiovascular death and stroke. In this modelling approach, the overall effect of a treatment on the outcome is decomposed into a direct effect and a pathway via a potential mediator. This pathway is determined by the effect of the treatment on the mediator and the effect of the mediator on the outcome, which may depend on the treatment group (treatment–mediator interaction). Thus, it may be that the mediator itself modifies the treatment effect when it is present. The ongoing treatment may be more (or less) advantageous for patients depending on the mediator, i.e. the mediator may moderate the treatment effect. Potential mediators of treatment effect are thus variables that are affected by the treatment and associated with the outcome. This four-way decomposition allows to estimate different effects:

- The total effect (TE) is an estimate of the treatment effect on the outcome, adjusted for potential confounders, taking the considered mediators into regard.
- The natural direct effect estimates the adjusted treatment effect on the outcome not caused by changes in the mediator.
- The controlled direct effects [CDE(m)] at Level m quantifies the adjusted treatment effect on the outcome, with the mediator being fixed at a certain value.
- The proportion eliminated [PE = (TE-CDE(m))/TE] is a measure that reflects the importance of a mediator for the explanation of the treatment effect if moderation effects are additionally taken into regard.

The outcome of interest in the mediation model is the time to the first primary endpoint occurring after the 12-month visit. We estimated the treatment and the mediator effect on the outcome using an accelerated failure time regression with an exponential distribution allowing to convert acceleration effects to hazard ratios (HRs). We selected the first in-person follow-up visit at 12 months (12-month follow up) as the survey time of potential mediators. All patients who had experienced the first primary endpoint by this time, who died or who had withdrawn their consent by then, were excluded from the mediation analysis. Later survey times would have led to a further reduction of the analysable sample and a loss of statistical power.

First, we examined all clinically meaningful parameters captured at 12-month follow up to determine the extent to which they were influenced by treatment. This was done by testing the difference in the 12-month measurement between randomized groups using adjusted mixed linear, mixed logistic, mixed ordinal (with site as random effect), or multinomial logistic regression, where appropriate. All models were further adjusted for the respective baseline measurement, if available. From the list of these potential mediators, we selected all variables with significant differences between the treatment groups to perform separate mediation analyses in a second step. Parameters that are a direct reflection of the intervention, e.g. echocardiography (ECG) changes reflecting antiarrhythmic drug therapy or complications of AF ablation, were not carried forward. The results of the mediation analyses are presented as HR with corresponding 95% CI of a landmark analysis with the first in-person follow-up visit at 12 months as the starting point.

To determine the effects of AF ablation on the primary outcome, we performed two further analyses studying ablation as a time-dependent variable.

### Objective 2

In a first step, we investigated the effect of ablation in the total study population using a Cox model with a time-dependent variable for ablation and an interaction term between treatment group and ablation to consider that the indication for an ablation was not the same in the two random groups. In this model, we also included all two-way interactions between adjusting baseline covariates and treatment group taking into account that the covariates may act differently in the random groups. We did a backwards selection (selection criterion *P* < 0.01) using likelihood ratio tests. As no interaction with treatment group showed a *P*-value <0.01, the adjusting baseline covariates remained in the model as main effects.

To describe the ablation effect within the ERC and usual care group, the respective contrasts of the two-way interaction between ablation and treatment groups were estimated and presented as HR with corresponding 95% CI.

### Objective 3

In a second step, we examined if there is a difference between early (delivered within 8 weeks after randomization) or late ablation (delivered >8 weeks after randomization). This analysis was restricted to the ERC patients only because the predominant indication for ablation differed between the random groups and there were only nine patients with early ablations in the usual care group. We used a Cox model with two time-dependent covariates. This analysis compared all periods after early ablation or late ablation with all periods without ablation, controlling for patient characteristics. Results are presented as HR together with 95% CI.

## Results

### Mediators of early rhythm control leading to improved cardiovascular outcomes (Objective 1)

**Figure 1:**
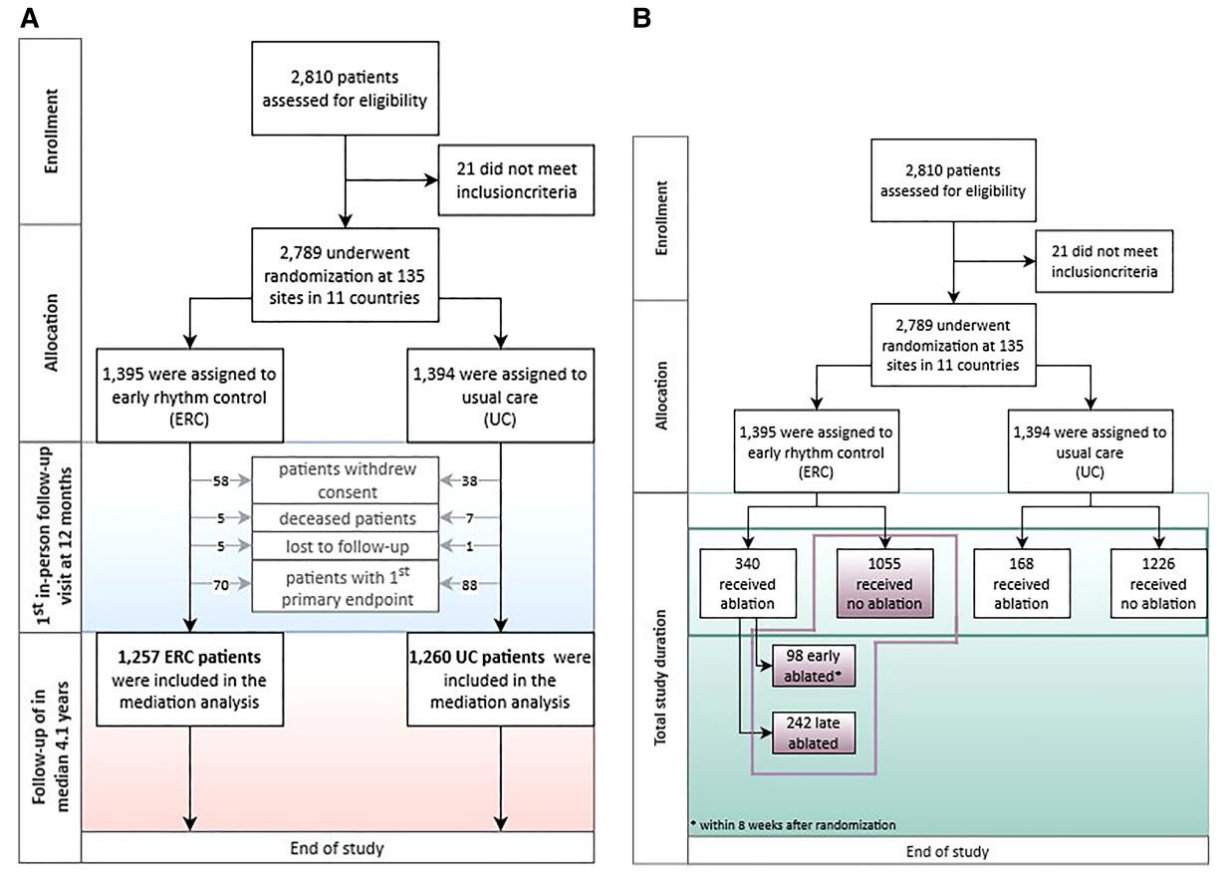
CONSORT diagram of the analyses. (*A*) Objective 1: Population analysis for the mediator analysis at the 12-month visit and follow-up time for future events (landmark analysis). (*B*) Objectives 2 and 3: Population analysis for the effect of atrial fibrillation ablation as a component of early rhythm control.

A total of 1257/1395 (90%) patients randomized to ERC and 1260/1394 (90%) patients randomized to usual care were seen at the 12-month visit without reaching a first primary outcome event (*Figure 1*). Analysis of all parameters captured at that visit identified 14 potential mediators of the treatment effect on the outcome (*Table 1*). The effects of ERC on these parameters at 12 months were determined. Systolic blood pressure, sinus rhythm at 12 months, and no recurrence of AF up to 12 months were significantly different between random groups (*Table 1*).

**Table 1:** Descriptive statistics of the potential mediators at 12 months by random group; restricted to patients without first primary endpoint and withdrawal of consent before first in-person follow-up visit at 12 months

		Random group		Total (*n* = 2517)	*P*-value
		Early rhythm control (*n* = 1257)	Usual care (*n* = 1260)		
**Body mass index (calculated) (kg/m²)**	Median (IQR)	28.4 (25.6–32.3)	28.7 (25.6–32.5)	28.6 (25.6–32.4)	0.817
	Missing	98 (8%)	102 (8%)	200 (8%)	
**Systolic blood pressure (mmHg)**	Median (IQR)	140 (127–150)	135 (125–150)	138 (125–150)	<0.001
	Missing	110 (9%)	98 (8%)	208 (8%)	
**Diastolic blood pressure (mmHg)**	Median (IQR)	80 (71–88)	80 (74–88)	80 (73–88)	0.971
	Missing	110 (9%)	98 (8%)	208 (8%)	
**Suspected acute coronary syndrome**		7/1178 (0.6%)	4/1185 (0.3%)	11/2363 (0.5%)	0.130
**AF recurrence(s) since last visit**	No	816 (69.3%)	718 (60.6%)	1534 (64.9%)	<0.001
	Yes, one time	122 (10.4%)	130 (11.0%)	252 (10.7%)	
	Yes, several times	190 (16.1%)	123 (10.4%)	313 (13.2%)	
	Still in AF^a^	50 (4.2%)	214 (18.1%)	264 (11.2%)	
	Missing	79 (6%)	75 (6%)	154 (6%)	
**Type of AF**	Paroxysmal	873 (77.5%)	733 (75.5%)	1606 (76.5%)	0.079
	Persistent or long-standing persistent	254 (22.5%)	238 (24.5%)	492 (23.5%)	
	Missing	130 (10%)	289 (23%)	419 (16%)	
**Overall symptom score (EHRA)**	EHRA I	875 (74.5%)	843 (71.1%)	1718 (72.8%)	0.216
	EHRA II	263 (22.4%)	302 (25.5%)	565 (23.9%)	
	EHRA III	36 (3.1%)	39 (3.3%)	75 (3.2%)	
	EHRA IV	1 (0.1%)	1 (0.1%)	2 (0.1%)	
	Missing	82 (7%)	75 (6%)	157 (6%)	
**Cardiac rhythm**	Sinus rhythm	977/1140 (85.7%)	763/1150 (66.3%)	1740/2290 (76.0%)	<0.001
**Heart rate (b.p.m.)**	Median (IQR)	62 (57–71)	64 (57–71)	63 (57–71)	0.461
	Missing	252 (20%)	481 (38%)	733 (29%)	
**Ventricular rate in AF (average of 10 intervals)**	Median (IQR)	80 (69–92)	81 (71–94)	81 (71–94)	0.161
	Missing	1123 (89%)	889 (71%)	2012 (80%)	
**Bundle branch block**		156/1103 (14.1%)	124/1124 (11.0%)	280/2227 (12.6%)	0.010
**AV nodal block**		203/1093 (18.6%)	151/1096 (13.8%)	354/2189 (16.2%)	<0.001
**INR value**	Median (IQR)	1.3 (1.1–2.1)	1.4 (1.1–2.1)	1.3 (1.1–2.1)	0.277
	Missing	469 (37%)	426 (34%)	895 (36%)	
**PT value (seconds)**	Median (IQR)	57 (32–88)	58 (33–87)	57 (32–87)	0.852
	Missing	673 (54%)	638 (51%)	1311 (52%)	

*P*-values of the treatment effect on the respective potential mediator, adjusted for baseline characteristics, and the respective baseline measurement, if one was available (there was no baseline measurement available for AF recurrence(s) since last visit).

IQR, interquartile range.

a No documented SR in between.

**Figure 2:**
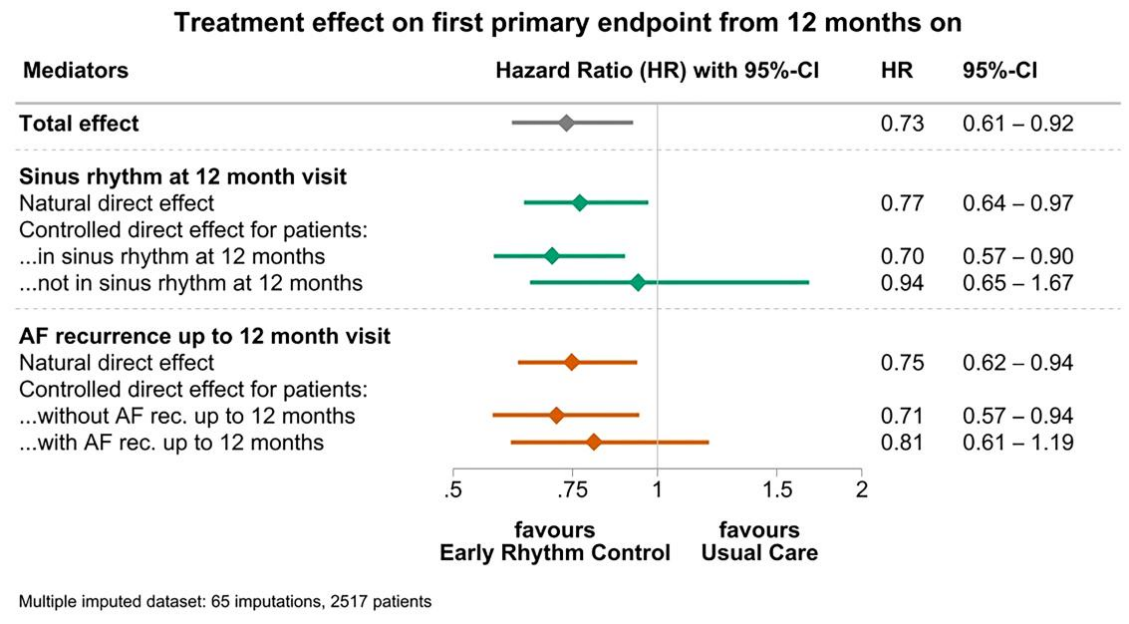
Strong mediating and moderating effect of sinus rhythm at 12 months on the first primary outcome of the EAST-AFNET 4 trial. The presence of sinus rhythm at 12 months explains about 81% of the effect of early rhythm control on the first primary outcome, a composite of cardiovascular death, stroke, or hospitalization for heart failure, or acute coronary syndrome. This can be appreciated in the first horizontal line in the graph (natural effect). There is hardly any effect of early rhythm control in patients who are not in sinus rhythm at the 12-month visit, visible in lack of a controlled direct effect in patients not in sinus rhythm at 12 months. Atrial fibrillation recurrence at any time up to the 12-month visit, in contrast, only explains 31% of the effect of early rhythm control, due to the small differences between the effects of the two subgroups (controlled effect in patients without AF recurrence and patients with AF recurrence). The analysis is adjusted for baseline characteristics that may confound the treatment effects on the mediator or the mediator effect on the outcome. Total effect indicates the adjusted treatment effect on the outcome; natural direct effect indicates the adjusted treatment effect due to the observed distribution of the mediator; controlled direct effect indicates the adjusted treatment effect for subgroups of patients with and without sinus rhythm or with and without atrial fibrillation recurrence at 12 months.

**Figure 3:**
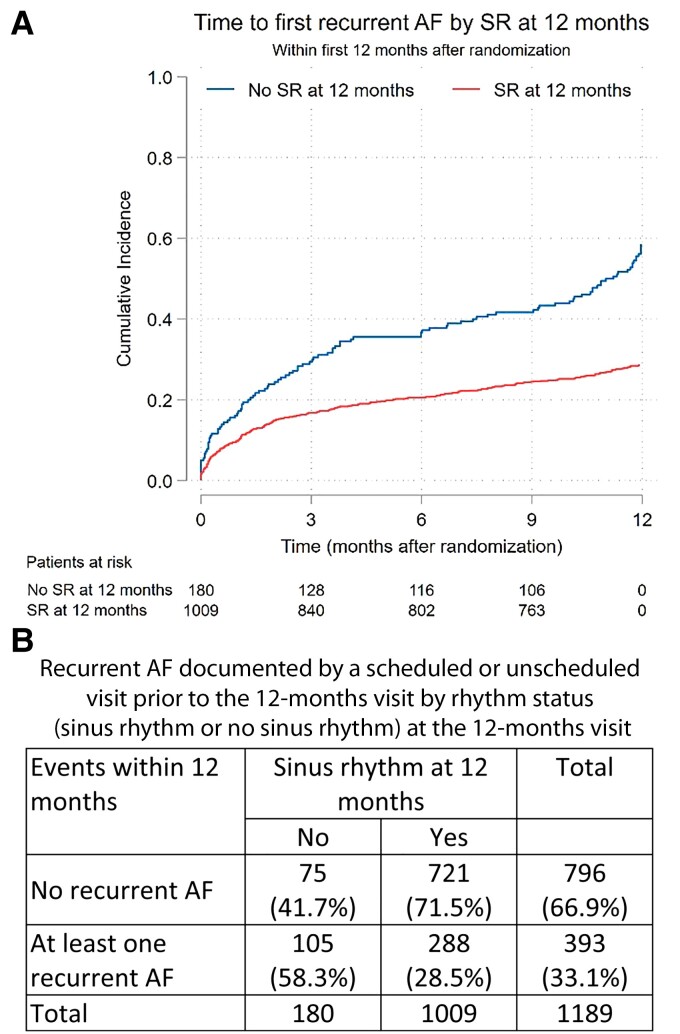
Time to recurrent atrial fibrillation and number of patients with recurrent atrial fibrillation in patients randomized to early rhythm control by rhythm status at 12 months. (*A*) The survival curves show the time to recurrent atrial fibrillation from randomization to 12 months in patients randomized to early rhythm control who were in sinus rhythm at 12 months and for patients who were not in sinus rhythm at 12 months. Patients who reached a primary outcome event or died in the first 12 months were not included. Recurrent atrial fibrillation was documented by triggered visits due to atrial fibrillation in the telemetric echocardiography or by an unscheduled visit due to clinically documented atrial fibrillation. (*B*) The tabulated data show the proportion of patients without recurrent atrial fibrillation and with recurrent atrial fibrillation who were in sinus rhythm at the 12-month visit: 58.3% (105/180) of patients not in sinus rhythm at 12 months had a scheduled or unscheduled visit due to recurrent atrial fibrillation, while only 28.5% (288/1009) of those in sinus rhythm at 12 months had such a visit.

To determine the effect of these mediators on outcomes, we conducted a landmark analysis evaluating all first primary outcome events after the 12-month visit. Early rhythm control reduced the first primary outcome from 12 months on up to the study end (median follow-up time 4.1 years, HR 0.73, 95% CI 0.61–0.92; *Figure 2*). Sinus rhythm at 12 months explained 81% of the treatment effect of ERC therapy compared with usual care during the remainder of follow up. In patients not in sinus rhythm at 12 months, ERC did not reduce further cardiovascular outcomes. Atrial fibrillation recurrence in the first 12 months of follow up only explained 31% of the treatment effect, systolic blood pressure at the 12-month visits only 10% (*Table 2*). For cardiovascular death and stroke, two key components of the primary outcome, similar effects were observed with larger CI due to smaller event numbers (*Table 2*). The key mediator ‘sinus rhythm at 12 months’ was partially correlated with recurrent AF in the first year (Spearman’s *ρ* = 0.59) and in a weaker way with paroxysmal AF (Spearman’s *ρ* = –0.32). As expected, recurrent AF was more common in patients who were not in sinus rhythm at the 12-month visit (*Figure 3*). None of the other mediators were correlated with sinus rhythm at 12 months (Spearman’s *ρ* = –0.22 to 0.13).

**Table 2:** Results of mediation analyses for three selected mediators and the three endpoints

Mediator/effect	Primary endpoint (*n* = 2517)				Cardiovascular death (*n* = 2635)				Stroke (*n* = 2616)			
	HR	95% CI	Proportion eliminated	95% CI	HR	95% CI	Proportion eliminated	95% CI	HR	95% CI	Proportion eliminated	95% CI
**Sinus rhythm at 12-month visit**												
Total effect	0.73	0.61–0.92			0.72	0.52–1.15			0.61	0.41–1.18		
Natural direct effect	0.77	0.64–0.97			0.78	0.57–1.23			0.67	0.46–1.26		
Controlled direct												
In sinus rhythm at 12 months	0.70	0.57–0.90			0.71	0.49–1.27			0.58	0.38–1.31		
Not in sinus rhythm at 12 months	0.94	0.65–1.67	0.81	–0.46 to 2.07	0.95	0.57–2.81	0.84	–0.95 to 2.64	0.91	0.52–3.79	0.85	–0.41 to 2.11
**AF recurrence up to 12-month visit**												
Total effect	0.74	0.62–0.93			0.73	0.53–1.15			0.63	0.42–1.25		
Natural direct effect	0.75	0.62–0.94			0.74	0.54–1.16			0.65	0.44–1.26		
Controlled direct												
Without AF rec. up to 12 months	0.71	0.57–0.94			0.71	0.48–1.35			0.61	0.37–1.75		
With AF rec. up to 12 months	0.81	0.61–1.19	0.31	–0.64 to 1.26	0.79	0.51–1.68	0.26	–1.37 to 1.89	0.73	0.47–1.65	0.26	–1.37 to 1.89
**Systolic blood pressure at 12-month visit** ^a^												
Total effect	0.72	0.60–0.91			0.75	0.55–1.18			0.73	0.48–1.46		
Natural direct effect	0.72	0.59–0.91			0.76	0.56–1.20			0.73	0.49–1.48		
Controlled direct												
At 135 mmHg^b^ at 12 months	0.72	0.60–0.91			0.76	0.56–1.19			0.71	0.48–1.32		
At 139 mmHg^c^ at 12 months	0.74	0.62–0.93	0.10	–0.06 to 0.26	0.74	0.55–1.17	–0.04	–0.31 to 0.23	0.67	0.47–1.20	–0.30	–1.14 to 0.54

The analyses are adjusted for baseline characteristics that may confound the treatment effects on the mediator or the mediator effect on the outcome. Total effect indicates the adjusted treatment effect on the outcome; natural direct effect indicates the adjusted treatment effect due to the observed distribution of the mediator; controlled direct effects indicate the adjusted treatment effect for subgroups of patients at a specified value of the mediator; and proportion eliminated is the proportion of the total effect that is due to variations of the mediator.

a Additionally adjusted for systolic blood pressure at baseline.

b The median value of the UC group at 12 months.

c The median value of the ERC group at 12 months.

### Effect of atrial fibrillation ablation (Objective 2)

**Figure 4:**
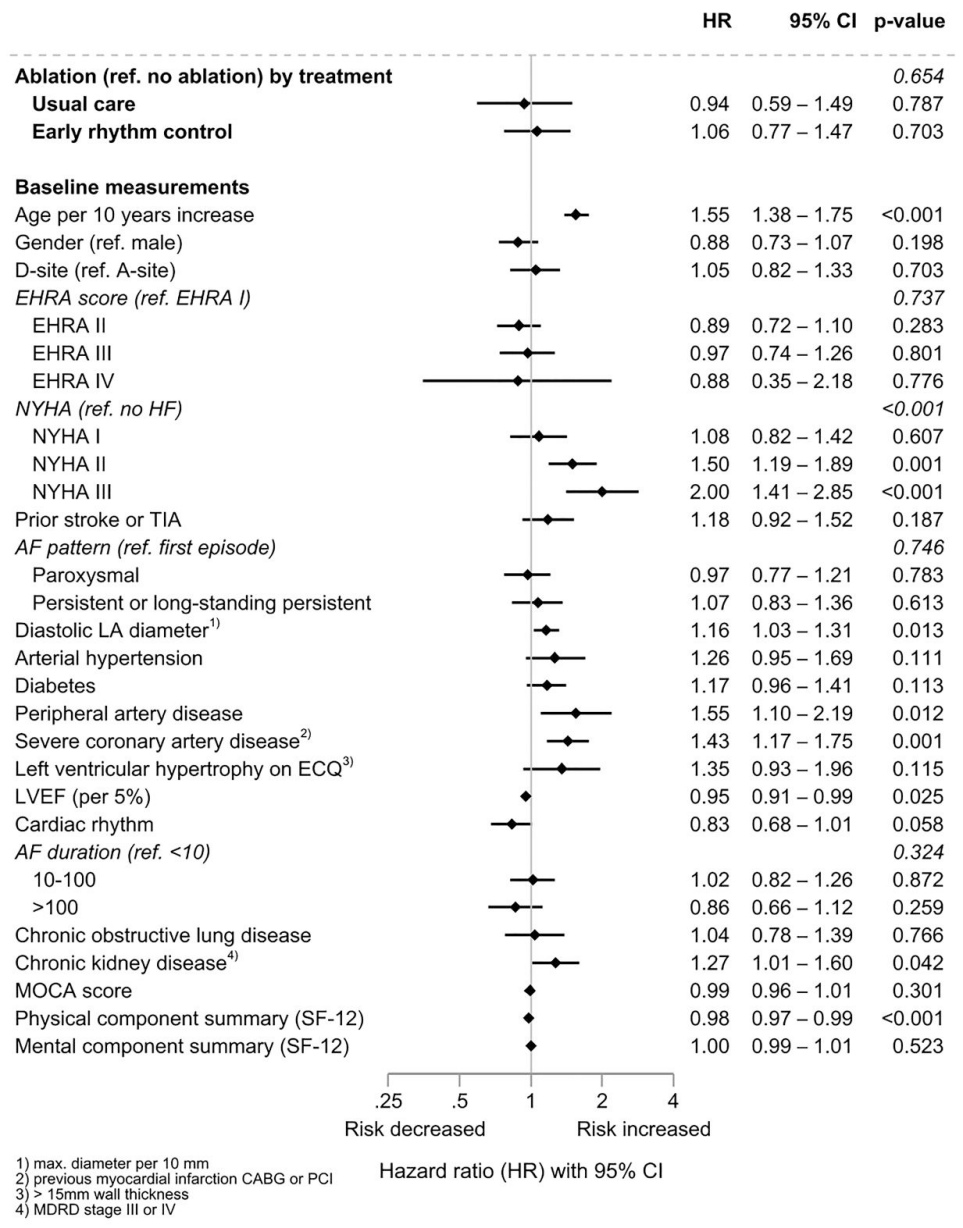
Association of atrial fibrillation ablation and primary outcome for all patients: results of an adjusted Cox model with ablation as a time-dependent predictor and its interaction with treatment, and random effect for centre (*n* = 2789, events = 565).

Patients who underwent AF ablation were younger than patients treated without AF ablation (*Table 3*). The use of AF ablation did not affect the primary outcome of the trial (*Figure 4*). Older age, presence of heart failure, peripheral or severe coronary artery disease, and a lower physical component of the SF-12 score had clear effects on the primary outcome (*Figure 4*, *Table 4*).

**Table 3:** Baseline demographic and clinical characteristics of all patients according to ablation or no ablation by treatment group. Patients summarised as “ablation” underwent atrial fibrillation ablation at any time point during the follow-up.

		Ablation			No ablation		
		ERC (*n* = 340)	UC (*n* = 168)	Total (*n* = 508)	ERC (*n* = 1055)	UC (*n* = 1226)	Total (*n* = 2281)
Age (years)	Median (IQR)	68 (62–73)	68 (64–72)	68 (63–73)	72 (66–77)	72 (66–77)	72 (66–77)
	≥ 75 years	54 (15.9%)	18 (10.7%)	72 (14.2%)	349 (33.1%)	391 (31.9%)	740 (32.4%)
Male sex		183 (53.8%)	89 (53.0%)	272 (53.5%)	567 (53.7%)	657 (53.6%)	1224 (53.7%)
Weight (kg)	Median (IQR)	84 (75–98)	82 (73–96)	84 (74–97)	80 (72–94	84 (72–95)	83 (72–95)
	Missing	2 (0.6%)		2 (0.4%)	5 (0.5%)	5 (0.4%)	10 (0.4%)
**AF characteristics**							
Type of AF	First episode	110 (32.4%)	57 (33.9%)	167 (32.9%)	418 (39.8%)	463 (37.8%)	881 (38.7%)
	Paroxysmal	110 (32.4%)	56 (33.3%)	166 (32.7%)	391 (37.2%)	437 (35.6%)	828 (36.4%)
	Persistent or long-standing persistent	120 (35.3%)	55 (32.7%)	175 (34.4%)	242 (23.0%)	326 (26.6%)	568 (24.9%)
	Missing				4 (0.4%)		4 (0.2%)
Heart rhythm	Atrial fibrillation or atrial flutter	193 (56.8%)	70 (41.7%)	263 (51.8%)	434 (41.4%)	580 (47.3%)	1014 (44.6%)
	Sinus rhythm and pacing	147 (43.2%)	98 (58.3%)	245 (48.2%)	615 (58.6%)	645 (52.7%)	1260 (55.4%)
	Missing				6 (0.6%)	1 (0.1%)	7 (0.3%)
Duration of AF history at baseline (days)	Median (IQR)	42 (6–123)	39 (7–140)	41 (6–136)	34 (6–108)	36 (5–106)	35 (6–107)
	Missing				2 (0.2%)	1 (0.1%)	3 (0.1%)
Overall symptom score (EHRA)	EHRA I (asympt.)	99 (31.1%)	38 (24.2%)	137 (28.8%)	296 (30.0%)	368 (31.4%)	664 (30.8%)
	EHRA II	156 (49.1%)	84 (53.5%)	240 (50.5%)	510 (51.7%)	608 (51.9%)	1118 (51.8%)
	EHRA III	62 (19.5%)	33 (21.0%)	95 (20.0%)	168 (17.0%)	184 (15.7%)	352 (16.3%)
	EHRA IV	1 (0.3%)	2 (1.3%)	3 (0.6%)	13 (1.3%)	11 (0.9%)	24 (1.1%)
	Missing	22 (6%)	11 (7%)	33 (6%)	68 (6%)	55 (4%)	123 (5%)
Previous pharmacological or electrical cardioversion		143/334 (42.8%)	83/168 (49.4%)	226/502 (45.0%)	403/1030 (39.1%)	460/1221 (37.7%)	863/2251 (38.3%)
LA diastolic diameter (max diameter) (mm)	Median (IQR)	44 (40–49)	44 (39–49)	44 (40–49)	42 (38–48)	43 (39–48)	43 (38–48)
	Missing	51 (15%)	25 (15%)	76 (15%)	141 (13%)	165 (13%)	306 (13%)
**Concomitant conditions**							
Prior stroke or transient ischaemic attack		42 (12.4%)	19 (11.3%)	61 (12.0%)	133 (12.6%)	134 (10.9%)	267 (11.7%)
MOCA total score	Median (IQR)	27 (24–29)	27 (25–29)	27 (24–29)	26 (23–28)	26 (23–28)	26 (23–28)
	Missing	8 (2.4%)	5 (3.0%)	13 (2.6%)	61 (5.8%)	48 (3.9%)	109 (4.8%)
At least mild cognitive impairment (MOCA < 26)		129 (38.9%)	55 (33.7%)	184 (37.2%)	453 (45.6%)	529 (44.9%)	982 (45.2%)
Arterial hypertension		307 (90.3%)	145 (86.3%)	452 (89.0%)	923 (87.5%)	1075 (87.7%)	1998 (87.6%)
Systolic blood pressure (mmHg)	Median (IQR)	138 (126–150)	135 (123–149)	136 (125–150)	133 (120–148)	137 (123–150)	135 (121–150)
	Missing	3 (0.9%)	1 (0.6%)	4 (0.8%)	6 (0.6%)	3 (0.2%)	9 (0.4%)
Diastolic blood pressure (mmHg)	Median (IQR)	80 (75–90)	80 (72–88)	80 (75–90)	80 (71–90)	80 (74–90)	80 (73–90)
	Missing	3 (0.9%)	1 (0.6%)	4 (0.8%)	6 (0.6%)	3 (0.2%)	9 (0.4%)
Stable heart failure (NYHA Stage II or LVEF < 50%)		88 (25.9%)	52 (31.0%)	140 (27.6%)	308 (29.2%)	350 (28.5%)	658 (28.8%)
Heart failure (NYHA classification)	No heart failure	219 (64.4%)	110 (65.5%)	329 (64.8%)	686 (65.3%)	804 (65.6%)	1490 (65.5%)
	I	49 (14.4%)	17 (10.1%)	66 (13.0%)	116 (11.0%)	149 (12.2%)	265 (11.6%)
	II	59 (17.4%)	32 (19.0%)	91 (17.9%)	196 (18.7%)	227 (18.5%)	423 (18.6%)
	III	13 (3.8%)	9 (5.4%)	22 (4.3%)	52 (5.0%)	46 (3.8%)	98 (4.3%)
	Missing				5 (0.5%)		5 (0.2%)
Severe coronary artery disease^a^		64 (18.8%)	29 (17.3%)	93 (18.3%)	179 (17.0%)	207 (16.9%)	386 (16.9%)
Peripheral artery disease		18 (5.3%)	4 (2.4%)	22 (4.3%)	45 (4.3%)	55 (4.5%)	100 (4.4%)
Diabetes		87 (25.6%)	30 (17.9%)	117 (23.0%)	264/1050 (25.1%)	313 (25.5%)	577/2276 (25.4%)
History of valve replacement		1 (0.3%)	2 (1.2%)	3 (0.6%)	10/1050 (1.0%)	10 (0.8%)	20/2276 (0.9%)
CHA2DS2-VASc score	Median (IQR)	3.0 (2.0–4.0)	3.0 (2.0–4.0)	3.0 (2.0–4.0)	3.0 (2.0–4.0)	3.0 (2.0–4.0)	3.0 (2.0–4.0)
Left ventricular hypertrophy on echocardiography^b^		18 (5.3%)	10 (6.0%)	28 (5.5%)	47 (4.5%)	57 (4.6%)	104 (4.6%)
Signs of valvular disease		160 (47.1%)	78 (46.4%)	238 (46.9%)	449/1049 (42.8%)	564/1223 (46.1%)	1013/2272 (44.6%)
Hb value (g/L)	Median (IQR)	145 (135–152)	143 (134–150)	144 (134–152)	142 (132–151)	142 (132–151)	142 (132–151)
	Missing	6 (1.8%)	2 (1.2%)	8 (1.6%)	19 (1.8%)	16 (1.3%)	35 (1.5%)
Chronic kidney disease (MDRD Stage III or IV)		29 (8.5%)	12 (7.1%)	41 (8.1%)	143 (13.6%)	167 (13.6%)	310 (13.6%)
GFR (MDRD formula)	Median (IQR)	76 (66–89)	75 (65–85)	76 (66–87)	76 (64–89)	76 (63–89)	76 (63–89)
	Missing	7 (2.1%)	1 (0.6%)	8 (1.6%)	20 (1.9%)	19 (1.5%)	39 (1.7%)
Chronic obstructive pulmonary disease		22 (6.5%)	10 (6.0%)	32 (6.3%)	73/1048 (7.0%)	104/1218 (8.5%)	177/2266 (7.8%)
Malignant diseases	No	316 (93.2%)	160 (95.2%)	476 (93.9%)	954 (91.1%)	1133 (92.5%)	2087 (91.9%)
	Yes, currently under therapy^c^	2 (0.6%)	0 (0.0%)	2 (0.4%)	6 (0.6%)	11 (0.9%)	17 (0.7%)
	Yes, not under current therapy^d^	21 (6.2%)	8 (4.8%)	29 (5.7%)	87 (8.3%)	81 (6.6%)	168 (7.4%)
	Missing	1 (0.3%)		1 (0.2%)	8 (0.8%)	1 (0.1%)	9 (0.4%)
Thyroid dysfunction (hypo- or hyperthyroidism)		43 (12.6%)	14 (8.3%)	57 (11.2%)	101/1050 (9.6%)	104 (8.5%)	205/2276 (9.0%)
**Medication at discharge**							
Missings					6 (0.6%)	1 (0.1%)	7 (0.3%)
Digoxin or digitoxin		14 (4.1%)	18 (10.7%)	32 (6.3%)	32 (3.1%)	67 (5.5%)	99 (4.4%)
Beta blockers		280 (82.4%)	146 (86.9%)	426 (83.9%)	778 (74.2%)	1045 (85.3%)	1823 (80.2%)
Calcium channel antagonists		114 (33.5%)	54 (32.1%)	168 (33.1%)	304 (29.0%)	393 (32.1%)	697 (30.7%)
ACE inhibitors or angiotensin II receptor blocker		242 (71.2%)	117 (69.6%)	359 (70.7%)	711 (67.8%)	862 (70.4%)	1573 (69.2%)
Oral anticoagulation (NOAC & VKA)		316 (92.9%)	152 (90.5%)	468 (92.1%)	951 (90.7%)	1098 (89.6%)	2049 (90.1%)
Mineralocorticoid receptor antagonist at discharge		27 (7.9%)	15 (8.9%)	42 (8.3%)	63 (6.0%)	77 (6.3%)	140 (6.2%)
Insulin		19 (5.6%)	1 (0.6%)	20 (3.9%)	43 (4.1%)	58 (4.7%)	101 (4.4%)
Oral antidiabetic		51 (15.0%)	22 (13.1%)	73 (14.4%)	177 (16.9%)	209 (17.1%)	386 (17.0%)
Diuretics		145 (42.6%)	60 (35.7%)	205 (40.4%)	414 (39.5%)	501 (40.9%)	915 (40.2%)
Antianginal drugs		4 (1.2%)	1 (0.6%)	5 (1.0%)	9 (0.9%)	10 (0.8%)	19 (0.8%)
Statin		162 (47.6%)	67 (39.9%)	229 (45.1%)	466 (44.4%)	501 (40.9%)	967 (42.5%)
Inhibitor of platelet aggregation		59 (17.4%)	26 (15.5%)	85 (16.7%)	170 (16.2%)	200 (16.3%)	370 (16.3%)

IQR, interquartile range.

a Previous myocardial infarction, CABG, or PCI.

b >15 mm wall thickness.

c With currently active disease manifestation.

d Without active disease manifestation.

**Table 4:** Baseline demographic and clinical characteristics of patients randomized to early rhythm control and undergoing no atrial fibrillation ablation, early atrial fibrillation ablation, or late atrial fibrillation ablation. Early atrial fibrillation ablation was defined as ablation as first-line rhythm control therapy used within the protocol-defined time period.

		No ablation (*n* = 1055)	Early ablation (*n* = 98)	Late ablation (*n* = 242)	Total (*n* = 1395)
Age (years)	Median (IQR)	72 (66–77)	67 (61–74)	68 (63–73)	71 (65–76)
	≥75 years	349 (33.1%)	21 (21.4%)	33 (13.6%)	403 (28.9%)
Male sex		567 (53.7%)	55 (56.1%)	128 (52.9%)	750 (53.8%)
Weight (kg)	Median (IQR)	82 (72–94)	85 (76.9–95)	84 (74–98)	82 (72–95)
	Missing	5 (0.5%)	1 (1.0%)	1 (0.4%)	7 (0.5%)
**AF characteristics**					
Type of AF	First episode	418 (39.8%)	26 (26.5%)	84 (34.7%)	528 (38.0%)
	Paroxysmal	391 (37.2%)	35 (35.7%)	75 (31.0%)	501 (36.0%)
	Persistent or long-standing persistent	242 (23.0%)	37 (37.8%)	83 (34.3%)	362 (26.0%)
	Missing	4 (0.4%)			4 (0.3%)
Heart rhythm	Atrial fibrillation or atrial flutter	4346 (41.4%)	556 (56.1%)	138 (57.0%)	627 (45.1%)
	Sinus rhythm and pacing	6156 (58.6%)	43 (43.9%)	104 (43.0%)	762 (54.9%)
	Missing	6 (0.6%)			6 (0.4%)
Duration of AF history at baseline (days)	Median (IQR)	34 (6–108)	45 (8–180)	42 (5–118)	36 (6–114)
	Missing	2 (0.2%)			2 (0.1%)
Overall symptom score (EHRA)	EHRA I (asymptomatic)	296 (30.0%)	22 (25.3%)	77 (33.3%)	395 (30.3%)
	EHRA II	510 (51.7%)	46 (52.9%)	110 (47.6%)	666 (51.0%)
	EHRA III	168 (17.0%)	19 (21.8%)	43 (18.6%)	230 (17.6%)
	EHRA IV	13 (1.3%)	0 (0.0%)	1 (0.4%)	14 (1.1%)
	Missing	68 (6%)	11 (11%)	11 (5%)	90 (6%)
Previous pharmacological or electrical cardioversion		403/1030 (39.1%)	42/96 (43.8%)	101/238 (42.4%)	546/1364 (40.0%)
LA diastolic diameter (max diameter) (mm)	Median (IQR)	42 (38–48)	45 (40–50)	44 (40–49)	43 (38–48)
	Missing	141 (13%)	19 (19%)	32 (13%)	192 (14%)
**Concomitant conditions**					
Prior stroke or transient ischaemic attack		133 (12.6%)	14 (14.3%)	28 (11.6%)	175 (12.5%)
MOCA total score	Median (IQR)	26 (23–28)	27 (24–29)	27 (24–29)	26 (23–28)
	Missing	61 (6%)	4 (4%)	4 (2%)	69 (5%)
At least mild cognitive impairment (MOCA < 26)		453 (45.6%)	39 (41.5%)	90 (37.8%)	582 (43.9%)
Arterial hypertension		923 (87.5%)	87 (88.8%)	220 (90.9%)	1230 (88.2%)
Systolic blood pressure (mmHg)	Median (IQR)	133 (120–148)	130 (122–145)	140 (129–150)	135 (122–150)
	Missing	6 (0.6%)		3 (1.2%)	9 (0.6%)
Diastolic blood pressure (mmHg)	Median (IQR)	80 (71–90)	80 (74–86)	80 (76–90)	80 (73–90)
	Missing	6 (0.6%)		3 (1.2%)	9 (0.6%)
Stable heart failure (NYHA Stage II or LVEF < 50%)		308 (29.2%)	28 (28.6%)	60 (24.8%)	396 (28.4%)
Heart failure (NYHA classification)	No heart failure	686 (65.3%)	60 (61.2%)	159 (65.7%)	905 (65.1%)
	I	116 (11.0%)	14 (14.3%)	35 (14.5%)	165 (11.9%)
	II	196 (18.7%)	20 (20.4%)	39 (16.1%)	255 (18.3%)
	III	52 (5.0%)	4 (4.1%)	9 (3.7%)	65 (4.7%)
	Missing	5 (0.5%)			5 (0.4%)
Severe coronary artery disease^a^		179 (17.0%)	16 (16.3%)	48 (19.8%)	243 (17.4%)
Peripheral artery disease		45 (4.3%)	5 (5.1%)	13 (5.4%)	63 (4.5%)
Diabetes		264/1050 (25.1%)	21 (21.4%)	66 (27.3%)	351/1390 (25.3%)
History of valve replacement		10/1050 (1.0%)	1 (1.0%)	0 (0.0%)	11/1390 (0.8%)
CHA2DS2-VASc score	Median (IQR)	3.0 (2.0–4.0)	3.0 (2.0–4.0)	3.0 (2.0–4.0)	3.0 (2.0–4.0)
Left ventricular hypertrophy on echocardiography^b^		47 (4.5%)	6 (6.1%)	12 (5.0%)	65 (4.7%)
Signs of valvular disease		449/1049 (42.8%)	53 (54.1%)	107 (44.2%)	609/1389 (43.8%)
Hb value (g/L)	Median (IQR)	142 (132–151)	146 (135–153)	143 (135–152)	142 (133–151)
	Missing	19 (2%)	1 (1%)	5 (2%)	25 (2%)
Chronic kidney disease (MDRD Stage III or IV)		143 (13.6%)	4 (4.1%)	25 (10.3%)	172 (12.3%)
GFR (MDRD formula)	Median (IQR)	75.7 (63.7–88.9)	77.6 (68.7–96.1)	75.0 (65.2–87.3)	75.7 (64.4–88.8)
	Missing	20 (2%)	3 (3%)	4 (2%)	27 (2%)
Chronic obstructive pulmonary disease		73/1048 (7.0%)	5 (5.1%)	17 (7.0%)	95/1388 (6.8%)
Malignant diseases	No	954 (91.1%)	93 (95.9%)	223 (92.1%)	1270 (91.6%)
	Yes, currently under therapy^c^	6 (0.6%)	0 (0.0%)	2 (0.8%)	8 (0.6%)
	Yes, not under current therapy^d^	87 (8.3%)	4 (4.1%)	17 (7.0%)	108 (7.8%)
	Missing	8 (0.8%)	1 (1%)		9 (0.6%)
Thyroid dysfunction (hypo- or hyperthyroidism)		101/1050 (9.6%)	12 (12.2%)	31 (12.8%)	144/1390 (10.4%)
**Medication at discharge**					
Missings		6 (0.6%)			6 (0.4%)
Beta blockers		778 (74.2%)	85 (86.7%)	195 (80.6%)	1058 (76.2%)
Calcium channel antagonists		304 (29.0%)	30 (30.6%)	84 (34.7%)	418 (30.1%)
ACE inhibitors or angiotensin II receptor blocker		711 (67.8%)	71 (72.4%)	171 (70.7%)	953 (68.6%)
Oral anticoagulation (NOAC & VKA)		951 (90.7%)	95 (96.9%)	221 (91.3%)	1267 (91.2%)
Mineralocorticoid receptor antagonist at discharge		63 (6.0%)	7 (7.1%)	20 (8.3%)	90 (6.5%)
Insulin		43 (4.1%)	5 (5.1%)	14 (5.8%)	62 (4.5%)
Oral antidiabetic		177 (16.9%)	14 (14.3%)	37 (15.3%)	228 (16.4%)
Diuretics		414 (39.5%)	42 (42.9%)	103 (42.6%)	559 (40.2%)
Antianginal drugs		9 (0.9%)	1 (1.0%)	3 (1.2%)	13 (0.9%)
Statin		466 (44.4%)	52 (53.1%)	110 (45.5%)	628 (45.2%)
Inhibitor of platelet aggregation		170 (16.2%)	17 (17.3%)	42 (17.4%)	229 (16.5%)

IQR, interquartile range.

a Previous myocardial infarction, CABG or PCI.

b >15 mm wall thickness.

c Or with currently active disease manifestation.

d And without active disease manifestation.

### Effect of atrial fibrillation ablation as a component of early rhythm control (Objective 3)

**Figure 5:**
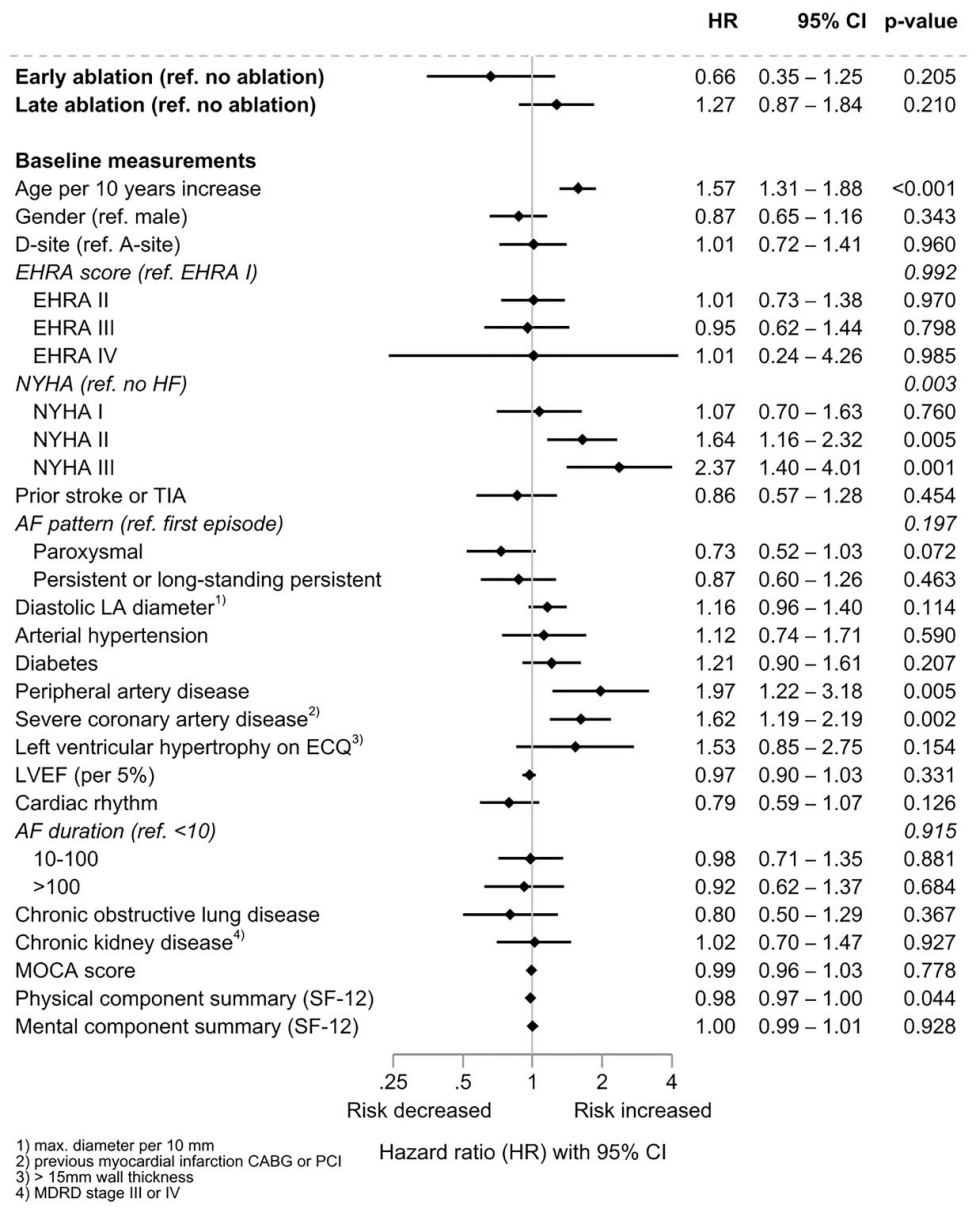
Association of early, late, or no atrial fibrillation ablation and primary outcome for patients randomized to early rhythm control: results of an adjusted Cox model with two time-dependent variables for early and late ablation as predictors and random effect for centre (*n* = 1395, events = 249).

A total of 340/1395 (24%) patients randomized to ERC therapy underwent AF ablation. Similar to the analysis in the entire population (*Figure 4*), older age, heart failure, and peripheral or severe coronary artery disease had clear effects on the primary outcome (*Figure 5*, all *P* ≤ 0.05). In an exploratory analysis of the timing of AF ablation, 98 patients underwent early AF ablation as first-line therapy within the protocol-specified period. Compared with non-ablated time intervals within patients randomized to ERC, cardiovascular outcomes appeared reduced in time intervals after early ablation within 8 weeks after randomization (HR 0.66, 95% CI 0.35–1.25; *Figure 5*). Atrial fibrillation ablation at a later time was associated with increased cardiovascular outcomes (HR 1.27, 95% CI 0.87–1.84 compared with non-ablated time intervals).

## Discussion

This prespecified, hypothesis-generating analysis of the EAST-AFNET 4 trial data set identified factors and mediators of ERC that were associated with reduced cardiovascular outcomes. Key findings are: (i) sinus rhythm at 12 months explained 81% of the treatment effect of ERC compared with usual care during the remainder of follow up; (ii) these analyses confirm that the effectiveness of the ERC therapy strategy tested in EAST-AFNET 4 relies on attaining sinus rhythm. Consequently, ERC therapy should aim for early and sustained restoration of sinus rhythm in patients with recently diagnosed AF to improve cardiovascular outcomes; and (iii) in the EAST-AFNET 4 trial setting where AF ablation was readily available when needed, the use of AF ablation was not associated with better outcomes than antiarrhythmic drug therapy (*Structured Graphical Abstract*). Future trials assessing the effectiveness and safety of early AF ablation in patients with cardiovascular comorbidities are warranted.

### Sinus rhythm at 12 months explains most of the effect of early rhythm control

Our main modelling analysis demonstrates that the presence of sinus rhythm at 12 months, the first follow-up interval with rhythm assessment in all patients, explains 81% of the outcome reduction achieved with ERC. This is an important mechanistic confirmation of the initial hypothesis of EAST-AFNET 4: restoring and maintaining sinus rhythm is the predominant effect of the ERC strategy in EAST-AFNET 4. Earlier trials comparing rate control only to rhythm control limited to antiarrhythmic drug therapy and cardioversion did not show reduced outcomes in patients randomized to rhythm control therapy.^11,12^ However, a modelling analysis of the AFFIRM data set also suggested that successful maintenance of sinus rhythm was associated with improved survival.^13^ The practice of stopping oral anticoagulation after apparently successful restoration of sinus rhythm may have led to worse outcomes in patients randomized to rhythm control therapy in AFFIRM.^13^ This factor was irrelevant in the present trial, as over 90% of all patients were on continued oral anticoagulation, irrespective of their rhythm status.^1,9^ A similar association of presence of sinus rhythm with better outcomes compared with patients who remained in AF was observed in the AF substudy of the DIAMOND trial.^14^ These prior analyses are in line with our analysis and underline the outstanding role of sinus rhythm for prognosis in AF patients. As a snapshot, the 12-month ECG follow up, even though it only provides a very crude estimate of AF burden and recurrent AF,^8,15,16^ identified patients in whom ERC therapy was not successful. These patients did not show reduced primary outcome events in our landmark analysis (*Figure 3*). Atrial fibrillation recurrence during the first year of follow up explained a smaller portion of the therapy effect. Broadly speaking, patients with recurrent AF are either patients in whom another attempt at rhythm control is successful (leading to sinus rhythm at 12 months), or patients in whom rhythm control remains futile (resulting in AF at 12 months). The former group is likely to see the beneficial effects of ERC, the latter probably not. This consideration can explain the weaker effect of recurrent AF on cardiovascular outcomes compared with attaining sinus rhythm at 12 months.

By identifying sinus rhythm during the initial follow up as the major mediator of the effectiveness of rhythm control therapy, our analysis provides a physiological rationale for ERC in routine care. The safety of ERC has been replicated in several analyses of large record data sets in South Korea,^17^ the USA,^5^ and in the UK BioBank.^6^ Consistent with our observation, randomization to the antiarrhythmic drug dronedarone was associated with reduced cardiovascular mortality and less ischaemic strokes (HR 0.66; 95% CI 0.45–0.96) in a subanalysis of the ATHENA trial (a placebo-controlled, double-blind, parallel arm trial to assess the efficacy of dronedarone 400 mg twice daily for the prevention of cardiovascular hospitalization or death from any cause in patients with AF/atrial flutter),^18^ in addition to reducing a primary outcome of death or cardiovascular hospitalization.^19^ Even the lack of effectiveness of dronedarone in the subsequent PALLAS study, where dronedarone therapy without restoration of sinus rhythm was associated with worse cardiovascular outcomes, can be aligned with our analysis.^20^ Based on this analysis, and consistent with explorative analyses of earlier trials, achieving sinus rhythm is the key mediator of ERC leading to reduced cardiovascular complications.

### Atrial fibrillation ablation as a component of early rhythm control

Atrial fibrillation ablation was readily available for all patients randomized to ERC therapy within EAST-AFNET 4.^1,9^ Early rhythm control was often initiated using antiarrhythmic drugs (1211/1395 patients; 87%). Overall, ablation was used in 340/1395 (24%) of the patients randomized to ERC in EAST-AFNET 4.^1,9^ This underpins that AF ablation was a necessary component of the ERC strategy but also highlights that most patients were treated without AF ablation. Atrial fibrillation ablation creates durable rhythm control in many patients and is more effective in maintaining sinus rhythm than antiarrhythmic drugs.^21,22^ Studies in selected patients with severe heart failure and AF suggested that AF ablation could improve outcomes^23^ and there is good evidence that AF ablation improves left ventricular function.^24^ These data led many to speculate that ablation-based rhythm control therapy would improve outcomes compared with antiarrhythmic drug-based rhythm control. Our exploratory analysis did not find that AF ablation was associated with better outcomes than antiarrhythmic drug therapy. Our analysis is supported by the neutral main finding of the CABANA trial, a randomized study comparing AF ablation to rhythm control based on antiarrhythmic drugs.^25^ An exploratory analysis of CABANA suggested that AF ablation may improve cardiovascular outcomes in young patients with fewer comorbidities.^26^ A post hoc subanalysis of the EAST-AFNET 4 trial, in contrast, demonstrated a strong beneficial effect of ERC in patients with multiple comorbidities, without a detectable effect of age.^27^ Given that AF ablation has so far mainly been evaluated in younger AF patients, dedicated clinical trials testing the effectiveness and safety of an AF ablation-dominated rhythm control strategy in patients with multiple comorbidities are needed, potentially utilizing simple ‘single-shot’ devices.^21,22,28^

### What can be learned for the management of patients with atrial fibrillation at 1 year after initiation of early rhythm control therapy?

A systematic ERC therapy strategy reduced outcomes in the EAST-AFNET 4 trial.^1,9^ This outcome-reducing effect of ERC was achieved by delivering therapy with relatively few complications. This main finding should in our view guide the management of patients presenting in AF after 12 months of rhythm control therapy. There will be patients in this group in whom further rhythm control therapy can be delivered with an acceptable safety profile, e.g. a first or recurrent AF ablation, or a combination of AF ablation and antiarrhythmic drugs.^29,30^ In others, in whom multiple therapies and experimental treatment combinations may be needed, a treatment strategy of rate control only may be advisable. Future trials may explore the best therapy for patients who are not in sinus rhythm after 1 year of ERC. Until such trials report, a careful balance of the expected effectiveness and safety of further rhythm control therapy seems warranted.

### Statistical considerations

An analysis using time-dependent covariates like the analysis presented here was also conducted within the AFFIRM trial.^13^ Differences in our analysis result from a different data structure: the date of AF ablation therapy was known while other potential mediators were only available at the two main follow-up visits (at 12 and 24 months). For these mediators, we used the causal mediation analysis approach to investigate the effect of them on the relationship between treatment and outcome. The classical approach introduced by Baron and Kenny^31^ and recently applied by Fitchett *et al*.^32^ was challenged by Valeri and Vanderweele^33^ who remarked that this approach does not allow causal interpretation in the presence of treatment–mediator interaction. The four-way decomposition used here allows the intended causal interpretation.

### Limitations

The presented analyses are not bias protected by randomization. Thus, an extended adjustment of covariates was required, but could not replace a randomization that was not possible by design in this study. Although we considered all factors measured at the 12-month visit in our mediation analysis, unmeasured confounders may explain the effect seen in patients who were in sinus rhythm at the 12-month visit in this analysis. Our analysis identifies sinus rhythm at 12 months as the dominant factor for future outcomes. The number of triggered, therapy-related visits was small in the EAST-AFNET 4 population, but we cannot exclude that a structured follow-up regimen contributed to differences in clinical outcomes seen in the trial. We used all available information to adjust for differences in baseline characteristics between the groups compared in these subanalyses. Still there may be differences that we have failed to adjust for. Our analysis may suffer from hidden biases and other unidentified confounders. Future randomized studies evaluating, e.g. ablation-based ERC strategies, are warranted. Furthermore, the described approach examined the effect of the mediators independently of each other, one at a time. The relationship between different mediators cannot be identified by use of observational mediation models as the mediators are not randomized to the treatment groups.^34^

## Conclusion

Successful rhythm control therapy, estimated by presence of sinus rhythm at 12 months after randomization, explains most of the reduction in cardiovascular outcomes achieved by ERC in the EAST-AFNET 4 trial. Based on these results, clinicians implementing ERC should aim for early and sustained restoration of sinus rhythm in patients with recently diagnosed AF and cardiovascular comorbidities. Further population-based investigations and clinical trials of AF management strategies may help to clarify the role of AF ablation and antiarrhythmic drug therapy for outcome reduction in patients with recently diagnosed AF and comorbidities.

## Data Availability

Data are made available upon request. Please email info@kompetenznetz-vorhofflimmern.de with a proposal of planned analyses.
